# Bayesian genomic selection: the effect of haplotype length and priors

**DOI:** 10.1186/1753-6561-3-s1-s11

**Published:** 2009-02-23

**Authors:** Trine Michelle Villumsen, Luc Janss

**Affiliations:** 1 University of Aarhus, Faculty of Agricultural Sciences, Department of Genetics & Biotechnology, Research Centre Foulum. DK-8830, Box 50, Tjele, Denmark; 2 University of Copenhagen, Faculty of Life Sciences, Department of Large Animal Sciences, 1870 Frb. C., Denmark

## Abstract

Breeding values for animals with marker data are estimated using a genomic selection approach where data is analyzed using Bayesian multi-marker association models. Fourteen model scenarios with varying haplotype lengths, hyper parameter and prior distributions were compared to find the scenario expected to give the most correct genomic estimated breeding values for animals with marker information only. Five-fold cross validation was performed to assess the ability of models to estimate breeding values for animals in generation 3. In each of the five subsets, 20% of phenotypic records in generation 3 were left out. Correlations between breeding values estimated on full data and on subsets for the "leave-out" animals varied between 0.77–0.99. Regression coefficients of breeding values from full data on breeding values from subsets ranged from 0.78–1.01. Single-SNP marker models didn't perform well. Correlations were 0.77–0.89 and predicted breeding values were biased. In addition the models seemed to over fit the genomic part of the variation. Highest correlations and most unbiased results were obtained when SNP markers were joined into haplotypes. Especially the scenarios with 5-SNP haplotypes gave promising results (distance between adjacent SNPs is 0.1 cM evenly over the genome). All correlations were 0.99 and regression coefficients were 0.99–1.01. Models with 5-SNP markers seemed robust to hyper parameter and prior changes. Haplotypes up to 40 SNPs also gave good results. However, longer haplotypes are expected to have less predictive ability over several generations and therefore the 5-SNP haplotypes are expected to give the best predictions for generations 4–6.

## Introduction

We present an approach for genomic selection (GS) where the data is analysed using Bayesian multi-marker association models. The analysed data is the QTLMAS XII common data set described in [1]. The aim is to get accurate genomic estimated breeding values (GEBV) for all individuals with marker information. We focus on how hyper and prior parameters and haplotype length affect the GEBV. A cross validation in generation 3 is used to evaluate the optimal model.

### Scenarios

Fourteen scenarios were analysed. The models had haplotype lengths of 1, 2, 5, 10, 20, and 40 SNPs. A two-mixture truncated normal distribution was used as prior for scaling factors that model the explained standard deviation per marker. We varied the proportion of markers to model the genetic effect and the prior parameter that determines the expected size of their scaling factors. The characteristics for each scenario are given in Table 1.

**Table 1:** Starting parameters and estimates for the scenarios.

**Scen.**	**Hap. Length**	***π*_1_**	**1^st ^step *σs*_1_**	**2^nd ^step *σs*_1_**	***σ* ^2^_*genom*_**	***σ* ^2^_*err*_**	***h* ^2^**	**Corr^a ^gen3**	**Reg^a ^gen3**
**1**	1	0.10	1.0	1.8	3.92	4.61	0.46	0.77	0.80
**2**	1	0.05	*	0.2	3.91	4.68	0.45	0.86	0.95
**3**	1	0.05	1.0	3.0	3.75	4.68	0.45	0.89	0.85
**4**	1	0.05	1.0	1.4	3.60	4.64	0.44	0.82	0.78
**5**	1	0.01	1.0	2.8	3.45	4.74	0.42	0.83	0.86
**6**	1	0.001	1.0	2.4	3.57	5.07	0.41	0.88	0.90
									
**7**	2	0.05	1.0	0.8	1.89	3.42	0.36	0.97	0.99
**8**	2	0.005	1.0	0.8	2.36	3.82	0.38	0.89	0.91
									
**9**	5	0.05	Optimize	0.17	1.55	3.09	0.33	0.99	1.01
**10**	5	0.01	Optimize	0.19	1.48	3.10	0.32	0.99	0.99
**11**	5	0.01	1.0	0.30	1.49	3.12	0.32	0.99	1.00
									
**12**	10	0.02	Optimize	0.23	1.50	3.08	0.33	0.98	0.99
**13**	20	0.04	Optimize	0.24	1.49	3.10	0.32	0.99	0.99
**14**	40	0.08	Optimize	0.24	1.53	3.05	0.33	0.97	0.99

^a ^GEBV of full data vs. GEBV of joint predicted data.

*Scenario 2 omits the 1^st ^step.

### Analysis model

The model uses phenotypes and genotypes from all individuals. Linkage phases of haplotypes are assumed to be known without error. The Bayesian model estimates allele substitution effects at all *m *markers, with *q*_*i *_alleles at marker *i*, as:

**y **= 1*μ *+ Σ_*i *_*φ*_*i *_Σ_*j *_**x**_*ij*_*β*_*ij *_+ **e**   *i *= 1, *m *and *j *= 1, *q*_*i*_

Here **y **is a vector of observations, *μ *is a general mean, **x**_*ij *_is a design vector indicating how many copies of the *j*^th ^allele of marker *i *are present in each observation, β_*ij *_is the allele substitution effect of the *j*^th ^allele of marker *i*, and **e **is a vector of model residuals, **e**~N(0, **I**σ_e_^2^). Allele effects are modelled as random with β_*ij*_~N(0,1) and *φ*_*i *_is a scaling factor that models the variance explained by *i*^th ^marker. The scaling factor can be interpreted as a standard deviation. A Bayesian variable selection method utilises a prior mixture distribution to select if certain model components are included in the model. To implement a selection on the level of markers, we chose a prior mixture distribution on the scaling factor *φ*_*i*_. The specification of the remaining prior distributions is:

$$\begin{array}{l} \mu \sim \text{constant}\\ \sigma_{\text{e}}^{2}\sim \text{constant}\\ \varphi_{i}\sim \{\begin{array}{cc} \pi_{\text{0 }}N(0,\sigma_{s0}^{2}) & \\ \pi_{\text{1}}N(0,\sigma_{s1}^{2}), & \varphi_{i}>0,\pi_{1}=1-\pi_{0} \end{array} \end{array}$$

The prior mixture distribution for scaling factors *φ*_*i *_follows the Bayesian variable selection method proposed by [2] where a large portion (*π*_0_) of *φ*_*i*_'s is forced to come from a distribution with small variance σs_0_^2^, while only a small fraction (*π*_1 _= 1 - *π*_0_) of *φ*_*i*_'s is allowed to have big effects coming from a distribution with large variance σs_1_^2^. The model is augmented with mixture indicators **γ **= {γ_*i*_}, indicating whether the *i*^th ^scaling factor comes from the first component of the mixture (γ_*i *_= 0) or from the second component of the mixture (γ_*i *_= 1).

### Obtaining parameter estimates

A MCMC sampler was used to generate samples from the joint posterior distribution of the model parameters f(*μ*, **φ**, **β**, **γ**, σ_e_^2^|**y**). The fully conditional distributions for *μ*, *ϕ*_*i*_'s and β_*ij*_'s are all Normal and the sampling of *ϕ*_*i*_'s is an alternation between two two normal distributions. The conditional posterior distributions of the mixture indicators (*γ*) are Bernouilli [2] and the conditional posterior distribution for residual variance is scaled inverse chi-square [3]. For all parameters single-variate Gibbs samplers were implemented. To obtain genomic predictions, MCMC chains were run in two steps. Aim of the first step was to get a good estimate of the hyper parameter σs_1_. MCMC chains for the full data were run with 10,000 cycles of which 3,000 burn-in; parameter samples were saved for each 100^th ^cycle. In the second step GEBV were estimated with a fixed setting for σs_1 _and leaving out certain phenotypes (see "Cross validation" below). For the models with haplotype length >= 5 the optimal σs_1 _estimated from the first step were used as known σs_1_. For models with haplotype length 1–2, due to failure in estimating σs_1 _at the first step, best σs_1 _was assumed equal to the largest *ϕ*_*i *_obtained from the first step. MCMC chains were run with 10,000 cycles, of which 3,000 burn-in for haplotype length 1–2 and 5,000 cycles of which 2,000 burn-in for larger length of haplotypes.

### Genomic predictions

GEBV were estimated for all individuals with marker information. The GEBV were constructed in each MCMC cycle as functions of the scaling factors *φ*_*i *_and allele effects β_*ij*_. Let **x**_*ij*_^E ^be extended versions of the design vectors with allele information for all individuals, then posterior samples of GEBV (g*) for all these individuals are:

g* = Σ_*i *_φ_i_* Σ_*j *_**x**_*ij*_^E ^β_ij_*

where asterix (*) indicates samples from the posterior distribution. By constructing **g **as a function of joint (*φ*_*i*_, β_*ij*_) samples within the MCMC, the covariances between *φ*_*i *_and β_*ij *_are automatically taken into account, and also the posterior standard deviations of **g **can be obtained. The final estimate for GEBV is the posterior mean obtained as the mean of a set of g* samples. Predictive abilities of genomic prediction models were assessed as the correlation between GEBV from full data and GEBV from subsets for the "leave-out" animals.

### Cross validation

To evaluate combinations of hyper parameter, prior distribution and haplotype length a five-fold cross validation was performed. In each of five data subsets phenotypic records were discarded for 2 out of 10 full sibs in generation 3, equal to 300 discarded records in each subset. Each record was only discarded once, deleting the records for full sibs 1–2 in the first cross validation data set, the records for full sibs 3–4 in the second cross validation data set, etc. Assuming that ordering of full sibs is random within family; this is equivalent to a (stratified) random deletion of records. Only records in generation 3 were discarded, because this is closest to generations 4 to 6 where GEBV were estimated from marker information only. For each subset a genomic prediction was performed using the same best estimate of σs_1 _and number of cycles as for the genomic prediction on *full *data. From each subset GEBV of the 300 individuals with discarded phenotypic records were joined into a new dataset. This is called the "*joined predicted*" data. In the "*joined predicted*" dataset GEBV for all 1500 individuals in generation 3 were based on marker information only, because the GEBV for each individual was retrieved from an analysis in which its own data was discarded.

For generation 3 correlations between GEBV from the full and joined predicted dataset and regression coefficient of GEBV from full data on GEBV from joined predicted data was computed. The best scenario is the one with highest correlation and the regression closest to one.

## Results

Table 1 summarizes the starting parameters and the estimates in 14 scenarios. For all scenarios σs_0 _is 0.01. The scenarios differ in haplotype length and in the settings for *π*_1 _and *σs*_1 _parameters, or in some cases included an estimation of *σs*_1 _from the data. Genomic variance *(σ*^2^_*genom*_), error variance (*σ*^2^_*err*_) and heritability (*h*^2^) are presented. The correlation between GEBV from full and joined predicted data and the regression of GEBV from full data on GEBV from joined predicted data in generation 3 are also given.

For single marker models the correlations in generation 3 between GEBV obtained in full and joined predicted data are 0.77–0.89, highest for Scenario 3. For Scenario 2–4 the only differences are in the setting of σs_1_. The largest *ϕ*_*i *_of the markers was used as σs_1 _in Scenario 3 while the second largest was used in Scenario 4. In Scenario 2, a value close to the automatic optimized σs_1 _in the scenarios with marker haplotypes was used to test if results were improved. The regression coefficient is 0.78–0.95, which indicates some bias. The lowest bias is found in Scenario 2. Table 2 shows that correlations between GEBV (for individuals in generation 3) obtained for full data in the six scenarios are 0.68–0.94, indicating that models are sensitive to prior and hyper parameter settings. The presented single marker models do not perform well.

**Table 2:** Correlations of GEBV in generation 3 between scenarios for single-SNP models, based on full data.

**Scenario**	**1**	**2**	**3**	**4**	**5**	**6**
**1**	1.00	0.68	0.86	0.77	0.91	0.61
**2**		1.00	0.86	0.94	0.77	0.90
**3**			1.00	0.91	0.89	0.81
**4**				1.00	0.84	0.90
**5**					1.00	0.68
**6**						1.00

In the scenarios 7 and 8 with 2-marker haplotypes the only difference is the setting of *π*_1_. The estimates of variances are different, but heritabilities are similar. Correlation and regression coefficients are higher than for single-SNP analyses. The correlation between GEBV from full data in the two scenarios is 0.96. The scenarios with 2-SNP haplotypes perform better than single-SNP models.

Scenarios 9 to 11 with 5-SNP haplotypes perform similarly, independent of prior and hyper parameter settings. Scenario 11 tests whether a large *σs*_1 _(using the largest *ϕ*_*i *_from the first step) results in the same results as the optimized *σ*s_1_. Variances and heritabilities are almost the same, correlations and regression coefficients are almost one. In generation 3, the correlations between GEBV from full data in the three scenarios were all 0.99. The 5-SNP haplotypes fit data well and without bias, and is robust to changes in priors and hyper parameter.

Scenarios with 10, 20, and 40-marker haplotypes had similar optimal *σs*_1_, same variances and heritabilities. Correlations and regression coefficients are all close to one. All three models are expected to give good GEBV estimates. Table 3 shows that correlations between the GEBV estimated from the full data are 0.94–0.97.

**Table 3:** Correlations of GEBV in generation 3 between scenarios for 10, 20 and 40-SNP models, based on full data.

**Scenario**	**12**	**13**	**14**
**12**	1.00	0.97	0.94
**13**		1.00	0.96
**14**			1.00

## Discussion

In this study we evaluated a Bayesian genomic prediction model with different grouping of SNPs into haplotypes and different settings of hyper and prior parameters. We assess the predictive abilities of the models using a cross validation in generation 3, assuming that the model which reached the best predictions in generation 3 would also be the model with the best predictions for generations 4 to 6.

Single-SNP models did not perform well. As shown in the cross validation, the correlations for the single-SNP models are the lowest observed among the given scenarios and the regression coefficients indicate bias. This was also found by [4]. Also the variance components in the single SNP models indicate bias and incorrect fitting of the data, with larger genomic variance and larger residual variance. This indicates that there must be a negative covariance between genomic fits and residuals, which is a sign of over fit. Overall, the single SNP models appeared variable and difficult to setting optimal prior and hyper parameter. We show that results from the single SNP model are relatively sensible for the prior and hyper parameter settings, and estimation of the hyper parameter *σs*_1 _from the data also failed for the single SNP models, probably due to collinearity between SNPs. A possible explanation for poor performance of the single-SNP model is that markers may not be in complete linkage disequilibrium with a QTL. In order to improve the single SNP model, probably lower settings for the *σs*_1 _parameter are needed.

Models based on haplotypes of 5 SNPs and more gave much better results. For these models, regressions indicated absence of biases and estimates of variance components were better: residual variance was smaller, which indicates a better model fit, and the total fitted variance was close to the raw variance in the trait. In these models there is no numerical problem in estimating parameter *σs*_1 _from the data. Using the *σs*_1 _estimated from the data is expected to give accurate and unbiased predictions.

Models based on 5-marker haplotypes are expected to give the best estimates of GEBV in generation 4–6. In the evaluation shown here the 10 to 40-SNP models perform equally well, but it should be noted that this is based on predictions in generation 3. For predicting breeding values in generations 4–6, however, these larger haplotypes may lose predictive ability quicker as shown by [4]. Therefore we expect the 5 SNP haplotypes to retain the best predictive ability over the generations 4–6. For estimation of GEBV in generation 4–6, Scenario 11 performs best with a correlation on 0.99 and a regression of exactly 1.00. GEBV from Scenario 11 are submitted for the workshop.

For scenario 11 the correlation between GEBV and true breeding value (TBV) in generation 4–6 turned out to be 0.92; the regression of TBV on GEBV from the full data was 0.98 indicating a small overestimation of GEBV. The amount of variance in TBV explained by GEBV, R^2 ^was 0.84. Hence, the selected model predicted the data well and gave nearly unbiased GEBV. The results can be found in table 2 in [1].

The better results here from using haplotypes than single-SNP is different from [5] in which IBD probabilities between haplotypes were used and where the use of such haplotypes gave similar results as the use of single SNPs. All results are in table 2 in [1]. There are however a number of differences between our approach and [5] in the use of Linkage and Linkage Disequilibrium (LD) possibly resulting in differences in the use of population-wide and family-specific effects. Our results show that significant improvements can be made from using haplotypes and that it is worthwhile to further investigate the use of haplotypes for making genomic predictions.

## Conclusion

We expect the most correct GEBV to be estimated in Scenario11, because the correlation between full and joined data is 0.99 and the regression coefficient is 1.00. The haplotype models perform better than single-SNP models and are less sensitive to prior and hyper parameter settings.

## List of abbreviations used

GEBV: genomic estimated breeding values; GS: genomic selection; TBV: true breeding value.

## Competing interests

The authors declare that they have no competing interests.

## Authors' contributions

TMV carried out the analyses and drafted the manuscript. LJ helped to draft the manuscript and to interpret and present the results in the manuscript. Both TMV and LJ read and approved the final manuscript.
